# Supplementary material for: Cytochrome oxidase requirements in Bordetella reveal insights into evolution towards life in the mammalian respiratory tract
Source: PLoS Pathog. 2024 Jul 8;20(7):e1012084. doi: 10.1371/journal.ppat.1012084 (PMC11257404; doi:10.1371/journal.ppat.1012084)
Supplement: S1 Fig — (PDF) [file ppat.1012084.s003.pdf]

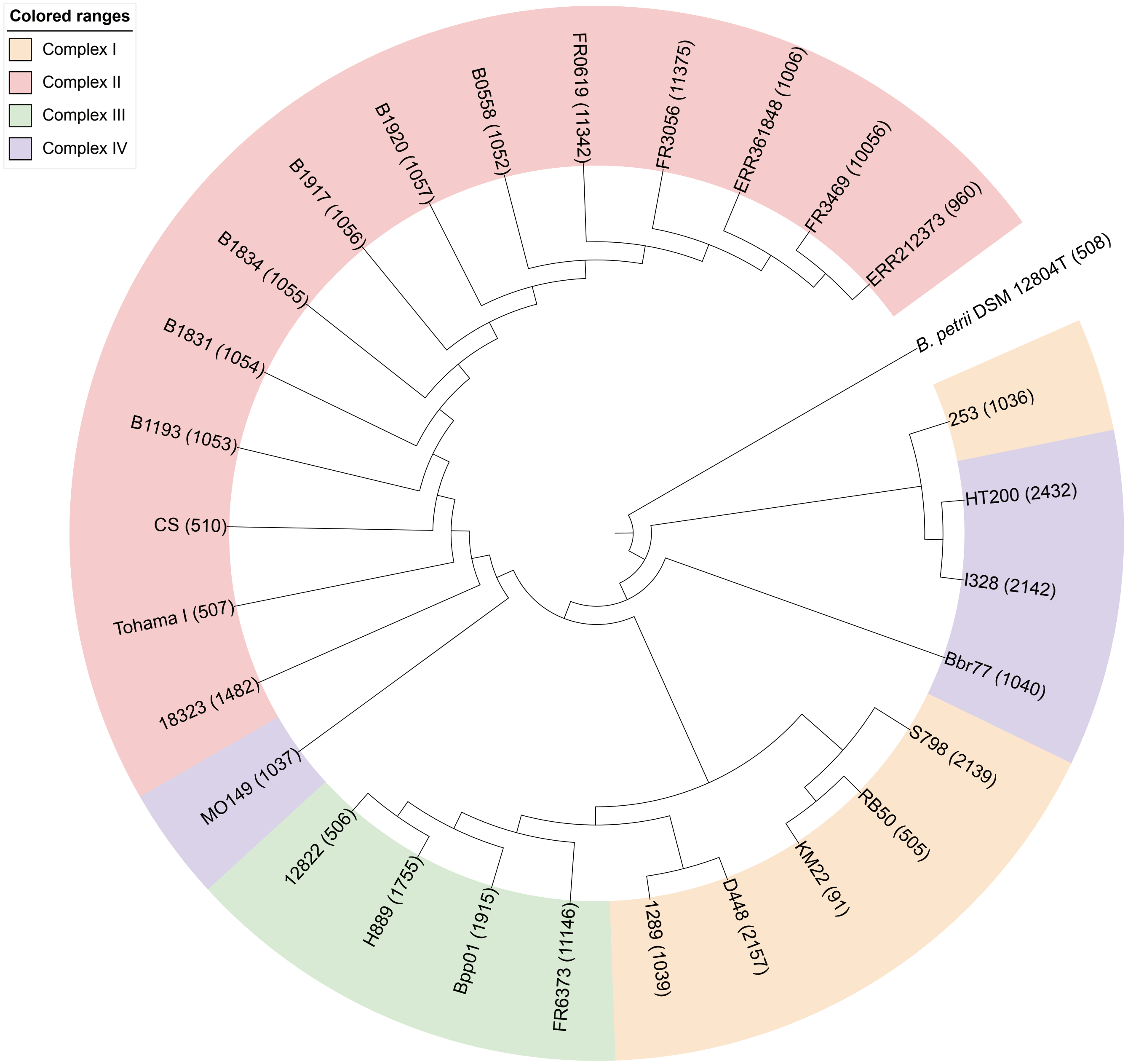

S1 Fig. Phylogenetic analysis of classical *Bordetella* strains. This tree was generated by comparing the concatenated multiple sequence alignments in the *cgMLST_genus* scheme in the BIGSdb-Pasteur genomic platform for Bordetella [1]. The tree was rooted on the branch leading to *B. petrii*. Leaves are labeled with the strain name and Institut Pasteur Bordetella cgMLST id. More information on the strains used can be seen in S1 Table. Tree created using Interactive Tree of Life (iTOL) v5 [2].

1. Bridel S, Bouchez V, Brancotte B, Hauck S, Armatys N, Landier A, et al. A comprehensive resource for *Bordetella* genomic epidemiology and biodiversity studies. Nature Communications. 2022;13: 3807. doi:10.1038/s41467-022-31517-8
2. Letunic I, Bork P. Interactive Tree Of Life (iTOL) v5: an online tool for phylogenetic tree display and annotation. Nucleic Acids Research. 2021;49: W293–W296. doi:10.1093/nar/gkab301
