# Supplementary material for: Cytochrome oxidase requirements in Bordetella reveal insights into evolution towards life in the mammalian respiratory tract
Source: PLoS Pathog. 2024 Jul 8;20(7):e1012084. doi: 10.1371/journal.ppat.1012084 (PMC11257404; doi:10.1371/journal.ppat.1012084)
Supplement: S3 Fig — (PDF) [file ppat.1012084.s005.pdf]

A.

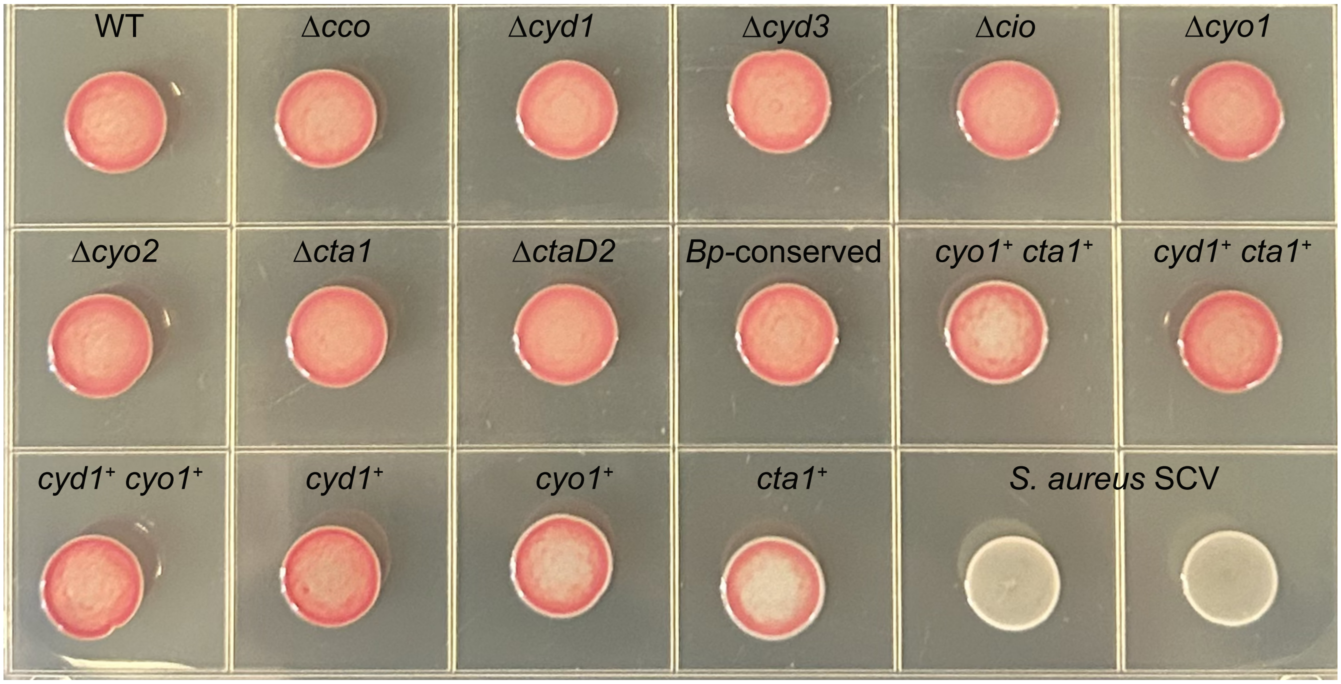

B.

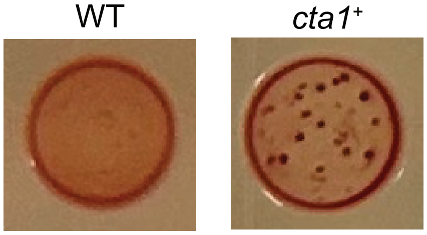

S3 Fig. All generated strains respire. TTC reduction after 24 hours of growth. Cultures were normalized to 1 OD_{600}/mL before spotting. When reduced, TTC undergoes an irreversible color change to red. A *Staphylococcus aureus menD* mutant (*S. aureus* SCV), which cannot respire, was included as a negative control. (A) Representative images of TTC reduction from 7 total biological replicates per strain. (B) In 4 out of 7 biological replicates, *cta1*$^{+}$ (right) had spots within the colony biofilm with a higher density of reduced TTC than WT (left). Raw data: https://doi.org/10.15139/S3/M7S90J
