# Supplementary figures and images for: Cytochrome oxidase requirements in Bordetella reveal insights into evolution towards life in the mammalian respiratory tract
Source: PLoS Pathog. 2024 Jul 8;20(7):e1012084. doi: 10.1371/journal.ppat.1012084 (PMC11257404; doi:10.1371/journal.ppat.1012084)

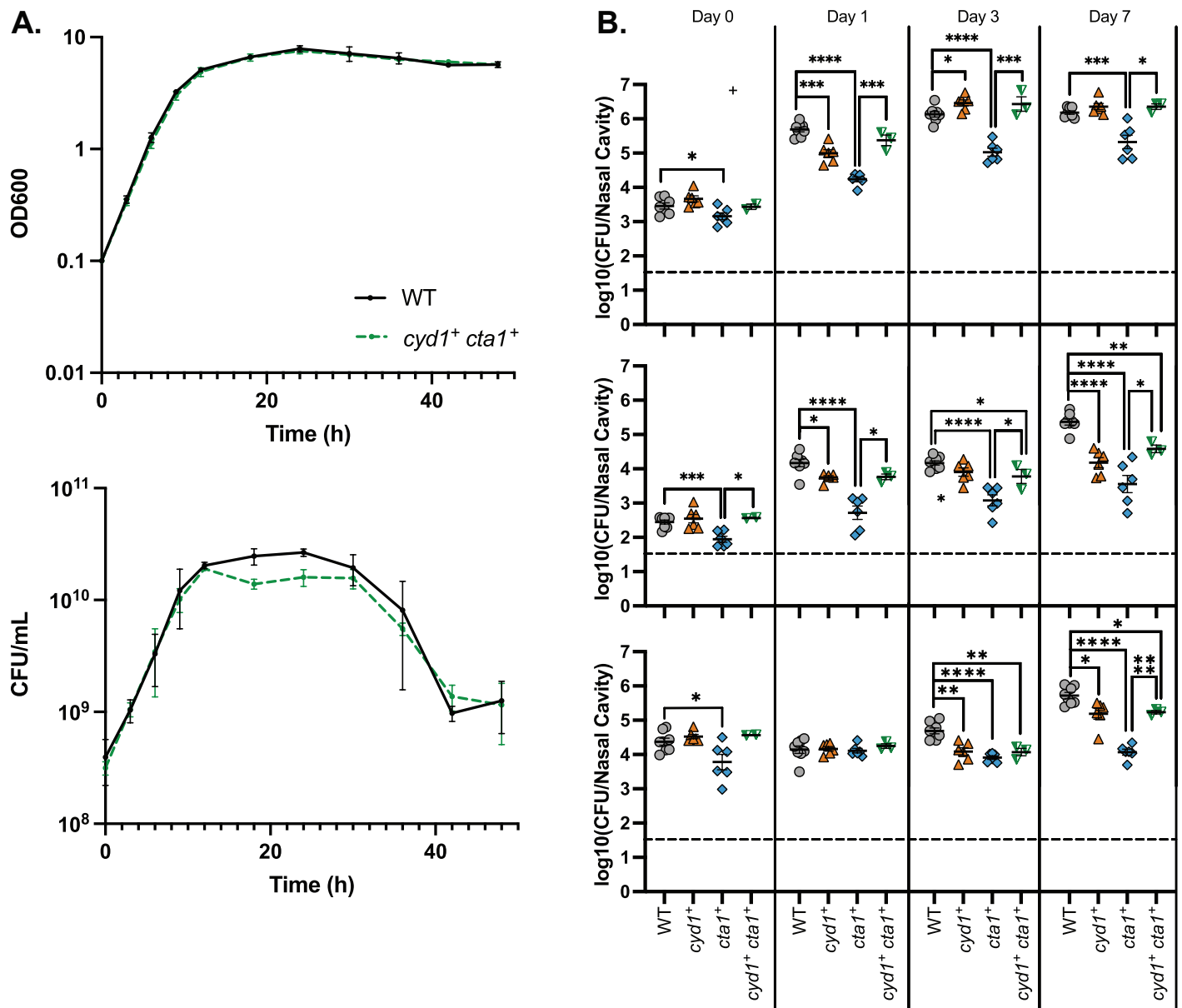

Supplement: S4 Fig — (PDF) [file ppat.1012084.s006.pdf]
