# Supplementary material for: Cytochrome oxidase requirements in Bordetella reveal insights into evolution towards life in the mammalian respiratory tract
Source: PLoS Pathog. 2024 Jul 8;20(7):e1012084. doi: 10.1371/journal.ppat.1012084 (PMC11257404; doi:10.1371/journal.ppat.1012084)
Supplement: S5 Fig — (PDF) [file ppat.1012084.s007.pdf]

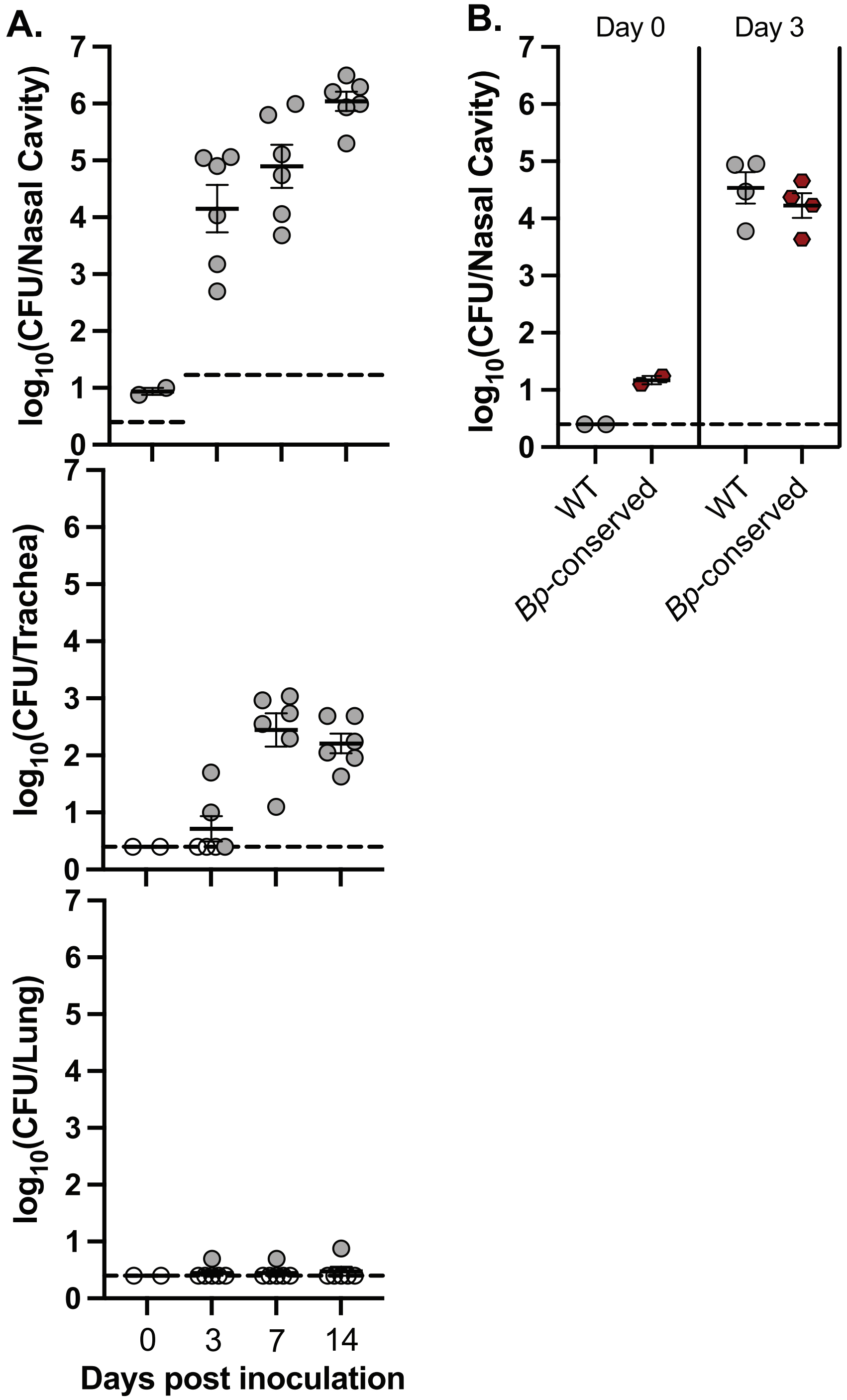

S5 Fig. The three cytochrome oxidases conserved in *B. pertussis* are sufficient for colonization in mice. (A) Bacterial burden over time within the nasal cavity (upper), trachea (middle), and right lung (lower) of mice infected with wild-type bacteria (WT, grey circles). Open circles represent tissue samples with no detectable bacteria. n=2 for day 0, n=6 for all other timepoints. (B) Bacterial burden over time within the nasal cavity of mice infected with wild-type bacteria (WT, grey circles) or a strain with only the cytochrome oxidase-encoding gene loci conserved in *B. pertussis* (*Bp*-conserved, maroon hexagon). n=2 for day 0, n=4 for day 3. Each point represents a single mouse. Dashed line represents the limit of detection. Open symbols represent samples below the limit of detection. Statistical significance was determined using unpaired Student's t-test; p-values are indicated when $p < 0.05$. Raw data: https://doi.org/10.15139/S3/EGWSWQ
