# Supplementary material for: Cytochrome oxidase requirements in Bordetella reveal insights into evolution towards life in the mammalian respiratory tract
Source: PLoS Pathog. 2024 Jul 8;20(7):e1012084. doi: 10.1371/journal.ppat.1012084 (PMC11257404; doi:10.1371/journal.ppat.1012084)
Supplement: S6 Fig — (PDF) [file ppat.1012084.s008.pdf]

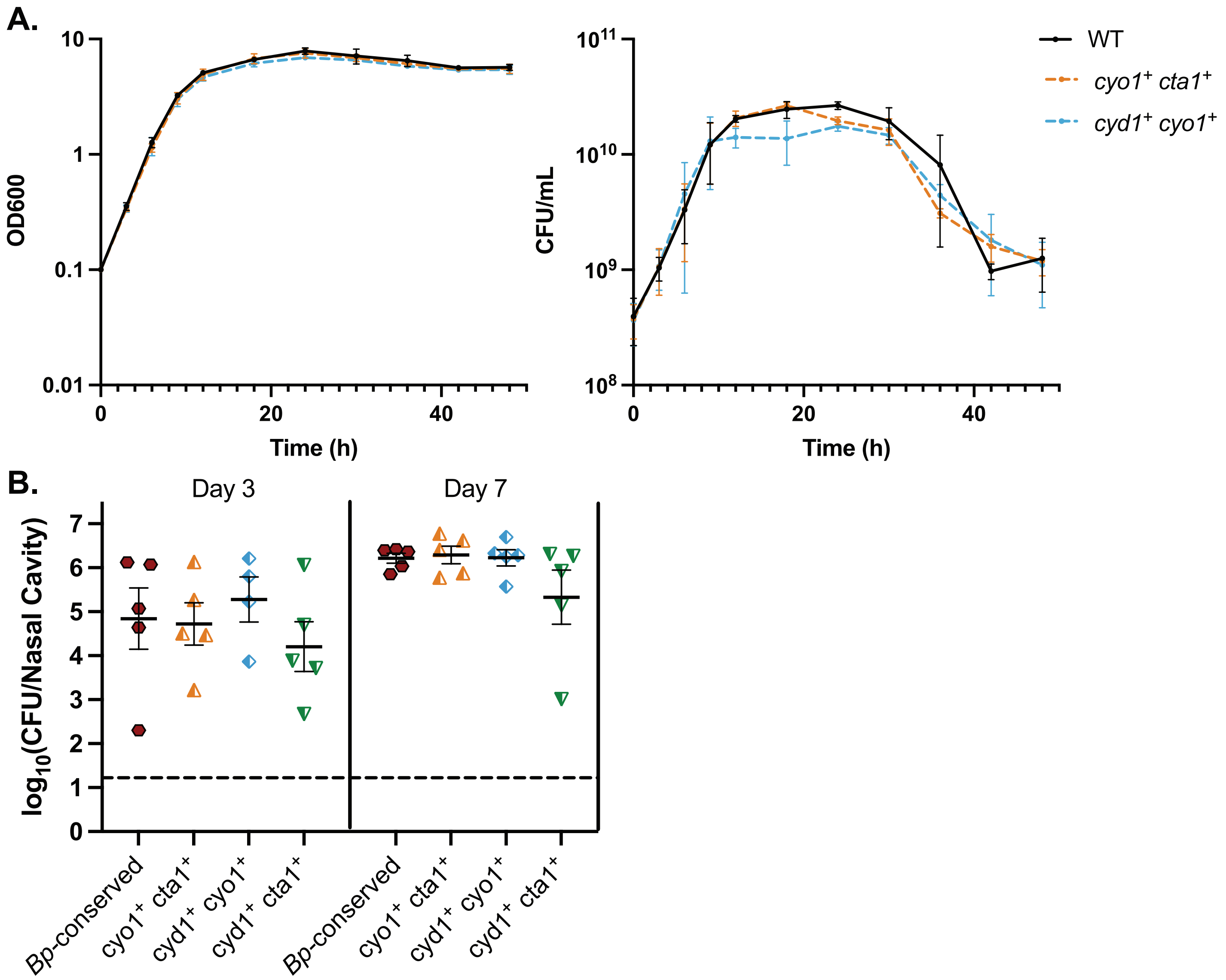

S6 Fig. No single cytochrome oxidase is required to establish infection. (A) Growth over time, measured via optical density (left) or CFU/mL (right), for strains with two cytochrome oxidase-encoding gene loci. The data for *cyd1*+ *cta1*+ can be found in S4A Fig. (B) Bacterial burden over time within the nasal cavity of mice infected with a strain with only the cytochrome oxidase-encoding gene loci conserved in *B. pertussis* (*Bp*-conserved, maroon hexagon), a strain with only *ctaCDFGE1* and *cyoABCD1* (*cta1*+ *cyo1*+, orange upright triangle), a strain with only *cydAB1* and *cyoABCD1* (*cyd1*+ *cyo1*+, blue diamond), or a strain with only *cydAB1* and *ctaCDFGE1* (*cyd1*+ *cta1*+, green upside-down triangle). Samples from 5 mice were collected at each timepoint for each strain. However, due to the natural microbiota of the nasal cavity, *B. bronchiseptica* could not always be enumerated due to contamination. Therefore, n=4 for *cta1*+ day 3. Each point represents a single mouse. Dashed line represents the limit of detection. Statistical significance was determined using unpaired Student's t-test; p-values are indicated when $p < 0.05$. Raw data: https://doi.org/10.15139/S3/X7T09R (S6A Fig); https://doi.org/10.15139/S3/MOWIZL (S6B Fig)
