# Supplementary material for: Cytochrome oxidase requirements in Bordetella reveal insights into evolution towards life in the mammalian respiratory tract
Source: PLoS Pathog. 2024 Jul 8;20(7):e1012084. doi: 10.1371/journal.ppat.1012084 (PMC11257404; doi:10.1371/journal.ppat.1012084)
Supplement: S2 Fig — (PDF) [file ppat.1012084.s004.pdf]

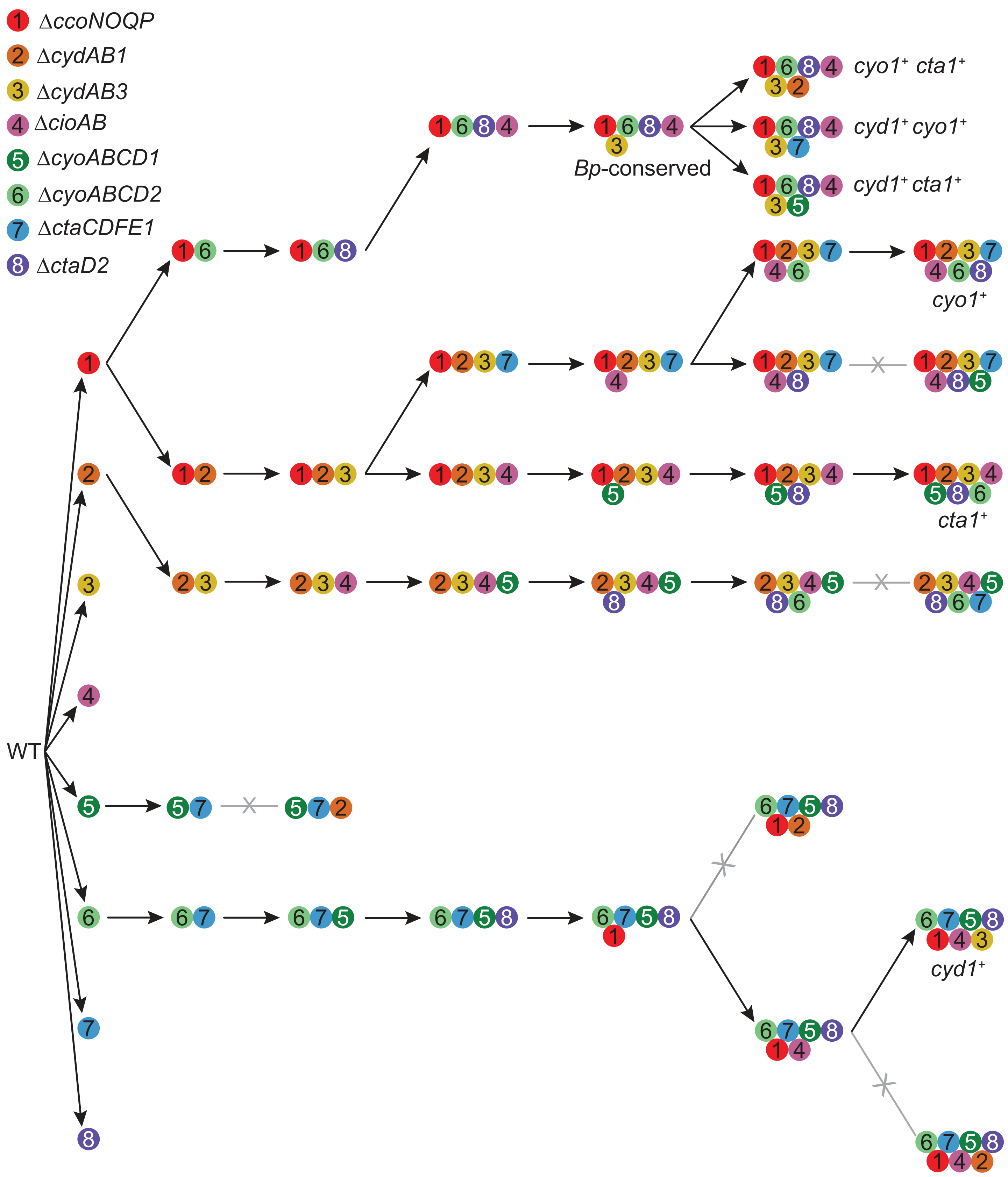

S2 Fig. Order of mutations added to generate strains. A full list of the strains used in this study can be found in Table 4. Numbers represent the in-frame deletion mutation added and also correspond to the mutations listed in Table 2. The names of strains have been added where applicable. Black arrows represent the successful deletion of a cytochrome oxidase-encoding gene locus. Gray crossed lines represent an allelic exchange that resulted only in wild-type revertants; at least 16 clones were screened for each.
